# Safety of High-Dose Botulinum Toxin Injections for Parotid and Submandibular Gland Radioprotection

**DOI:** 10.3390/toxins14010064

**Published:** 2022-01-17

**Authors:** Joerg Mueller, Thomas Langbein, Aditi Mishra, Richard P. Baum

**Affiliations:** 1 Department of Neurology, Vivantes Klinikum Spandau, Neue Bergstrasse 6, D-13585 Berlin, Germany; 2 Department of Nuclear Medicine, Technical University of Munich, Klinikum rechts der Isar, Ismaninger Strasse 22, D-81675 München, Germany; thomas.langbein@tum.de; 3 Department of Nuclear Medicine, Curanosticum Wiesbaden, Deutsche Klinik für Diagnostik, Aukammallee 33, D-65191 Wiesbaden, Germany; mishra@curanosticum.de (A.M.); baum@curanosticum.de (R.P.B.)

**Keywords:** high-dose botulinum toxin, salivary glands, radioprotection

## Abstract

Botulinum Toxin injections into salivary glands (SG) up to a total dose of 100 units IncobotulinumtoxinA (IncoA) represent the treatment of choice for sialorrhea. However, BTX might also protect SG against sialotoxic radioligand cancer therapies. The radioligand Actinium-225-PSMA effectively targets Prostate Cancer (PCa) metastases but inevitably destroys SG due to unintended gland uptake. A preliminary case series with regular-dose IncoA failed to reduce SG PSMA-radioligand uptake. We therefore increased IncoA dosage in combination with transdermal scopolamine until a clinically relevant SG PSMA-radioligand uptake reduction was achieved. Ten consecutive men with metastasized PCa refractory to all other cancer therapies received gradually increasing IncoA dosages as part of a compassionate use PSMA-radioligand-therapy trial. The parotid gland received six and the submandibular gland three injection points under ultrasound control, up to a maximum of 30 units IncoA per injection point. A maximum total dose of 250 units IncoA was applied with up to 170 units per parotid and 80 units per submandibular gland. Treatment was well tolerated and all side-effects were non-serious. The most frequent side-effect was dry mouth of mild severity. No dysphagia, facial weakness, chewing difficulties or systemic side-effects were observed. SG injections with IncoA up to a total dose of 250 units are safe when distributed among several injection-points under ultrasound control by an experienced physician. These preliminary findings lay the basis for future trials including BTX as major component for SG protection in established as well as newly emerging radioligand cancer therapies.

## 1. Background

In healthy adults, the parotid and submandibular glands account for 95% of the total salivary secretion. Botulinum Toxin (BTX) injections into these major salivary glands are considered the treatment of choice for chronic neurogenic sialorrhea [1]. Among the type-A botulinum toxin preparations, only IncobotulinumtoxinA (IncoA) is approved for the treatment of sialorrhea in the US and EU. The recommended total dose is 75 to 100 units IncoA, with up to 30 units given to each parotid and up to 20 units to each submandibular gland. Apart from neurological indications, the use of BTX as a salivary gland protective agent in cancer patients with normal salivary gland function might emerge as a promising new BTX-indication. Radioligand therapies against prostate or thyroid cancer frequently induce gland destruction due to unintended radioligand-uptake into the healthy salivary glands. Severe sialotoxicity represents the treatment-limiting side-effect of the highly effective prostate cancer radioligand therapy with Actinium-225-PSMA (Ac-PSMA) [2,3]. All previous attempts with various drugs including monotherapy with anticholinergics failed to overcome this therapy-limiting side-effect [3,4]. Patients with differentiated thyroid cancer treated with radioactive iodine (^131^I) are also at risk of permanent xerostomia. Selvakumar et al. reported that almost 50% of patients from a nationwide study in the Netherlands suffered from permanent salivary gland dysfunction with moderate to severe xerostomia in 35% at median 11 years following radioiodine therapy with ^131^I [5]. Salivary gland destruction is associated with severe xerostomia inducing a plethora of complications resulting in a significant reduction in quality of life.

A recent publication proved the relationship between salivary gland activity and salivary gland uptake of Prostate-Specific Membrane Antibody (PSMA) radioligands [6].

BTX reduces salivary gland activity in a dose-dependent manner with subsequent and fully reversible ductal and acinar apoptosis [7,8]. However, in a preliminary case series with regular-dose IncoA distributed across the submandibular and parotid glands we observed no reduction of Lutetium-144-PSMA (Lu-PSMA) radioligand uptake in any of the injected glands. Therefore, the present study intended to assess safety and tolerability of gradually increasing SG BTX-A doses required to achieve a clinically relevant PSMA-uptake reduction. Toxic Ac-PSMA salivary gland uptake correlates with the amount of gland destruction and clinical xerostomia [9] occurs when salivary gland function is reduced by about 50% [10]. Here, we report and discuss the preliminary safety data from 10 advanced prostate cancer (PCa) patients who received increasing IncoA dosages as part of a compassionate use PSMA-radioligand therapy trial. The oncological and Positron emission tomography (PET) outcome data will be reported elsewhere (manuscript in preparation).

## 2. Patients and Methods

10 consecutive men with a mean age of 68 years [range 53–80] were treated with increasing dosages of IncoA by means of a compassionate use trial. All men suffered from advanced metastasized PCa for whom all other treatment options had failed. All patients were refractory to previous surgery, external radiation, hormone- and various chemotherapies as well as to previous radioligand monotherapies with Lu-PSMA and thus scheduled for a compassionate use trial with a combined Lu-PSMA plus Ac-PSMA “tandem” radioligand therapy. Since Ac-PSMA therapy is known to induce immediate complete and irreversible salivary gland destruction as side-effect and no established gland protective therapy has been available, patients were offered BTX injections as part of the compassionate trial. All patients gave written informed consent following detailed explanation of the procedure and possible side-effects—in particular dysphagia. All procedures were conducted in accordance with the Declaration of Helsinki.

Measures of saliva production were performed prior to IncoA and after radioligand therapies. Unprovoked salivary flow was measured with four dental rolls placed in the orifice of the mouth and then retained there for five minutes. Provoked salivary flow was measured by chewing on a folded compress placed on the tongue for two minutes [11]. The difference in weight between the dry and wet rolls was calculated in grams (g) and both measures were added up.

Each 100 unit IncoA (Xeomin®, Merz Pharmaceuticals, Frankfurt, Germany) vial was reconstituted with 1 mL normal 0.9% saline using a 1 mL syringe. BTX was injected percutaneously (26 G, 25 mm needle length) into the salivary glands under ultrasound control using a GE 7.5 MHz Linear Array Ultrasound Probe (GE Health Care, Chicago, IL, USA). The parotid gland received six injection points (between-point distance around 10 mm, each injection point with the same dose). The submandibular gland received three injection points, each with the same dose. A maximum dose of 30 units IncoA per injection point was not exceeded.

All patients received IncoA-injections into two of the four main salivary glands (right parotid plus contralateral left submandibular gland). Injections were performed median 14 days [range 5–33 days] before tandem-PSMA radioligand therapy by a neurologist with more than 25 years of experience with BTX.

Patients were closely monitored by phone interviews and repeat clinical follow-up visits. Predefined adverse events of special interest were dry mouth, dysphagia, facial weakness, eyelid-drop, conjunctivitis, stomatitis, chewing and breathing difficulties as well as generalized weakness. Side-effects were recorded using a 0 to 3 scale (none/mild/moderate/severe). The concomitant permanent medication was recorded at the time of BTX injection.

## 3. Results

Table 1 summarizes BTX treatment details and side-effects for each patient. A maximum total dose of 250 units IncoA was applied with up to 170 units into a single parotid gland and 80 units IncoA into a single submandibular gland. Treatment was well tolerated and all side-effects were non-serious.

Mild to moderate injection pain was experienced by all patients and resolved immediately. Injection pain was more pronounced in the parotid glands. One injection-related local hematoma occurred in a patient with thrombocytopenia.

The most frequent BTX-related side-effect was dry mouth of mild severity. Patient 1 who received a total dose of 150 units IncoA reported a mild flu-like painful swallowing episode which lasted for one week and resolved without intervention. Patients 6 + 8 had pre-existing moderate xerostomia after previous chemo- and radiation therapies that remained unchanged (see Table 1).

No dysphagia, facial weakness, eyelid-drop, conjunctivitis, stomatitis, chewing or breathing difficulties were observed. No patient showed distant or systemic side-effects.

Follow-up: at the time of manuscript submission, four of the ten patients had completed two Ac-PSMA cycles and were scheduled for their third and fourth Actinium-225- Prostate Specific Membrane Antigen (Ac-PSMA) radiotherapy (RT). Combined unprovoked and provoked saliva production 16–20 weeks after the first IncoA injection resulted in a mean 29% [range 25–31%] loss (or mean 71% preservation) of saliva production as compared to around 60–70% salivary gland destruction following two cycles of unprotected Ac-PSMA [3].

Concomitant medication: the concomitant medication is summarized in Table 2. All patients received endocrine therapies and all patients used at least one pain medication constantly. Seven patients received additional transdermal scopolamine during a period of 72 h prior PSMA-radioligand therapy.

## 4. Discussion

To the best of our knowledge, these are the first data to report the safety and tolerability of high-dose BTX-A injections into the salivary glands up to a total dose of 250 units IncoA. The individual glands received four to five times the dose regularly used to treat sialorrhea. Notably, the injected dosage of 170 units IncoA (Xeomin^®^) into a single parotid gland was tolerated without any swallowing or chewing problems or systemic side-effects. Likewise, injected dosages of 80 units IncoA into a single submandibular gland did not result in swallowing problems. The latter result is of particular interest considering the glands proximity to the supra-hyoidal muscles critical for swallowing function. Our results also indicate that high-dose salivary gland treatment with IncoA under ultrasound guidance using a maximum of 30 units per injection point is not associated with clinically relevant local or systemic toxin spread. A mandatory requirement for the application of high-dose BTX into salivary glands is ultrasound-guidance in the hand of an experienced neurologist or Otolaryngologist. Previous studies demonstrated the superiority of ultrasound guided injections over anatomical landmark-oriented injections. A post-mortem study showed an almost doubled hit-rate of ultrasound-guided injections in the submandibular glands compared to free-hand injections [12]. The high error-rate even of experienced injectors might be attributable to the variance of submandibular gland size as well as to different inter- and intra-individual distances between gland and mandible [1,13].

It remains a matter of debate whether more than one injection point per gland improves outcome and/or reduces the risk of side-effects from local toxin spread. However, histological analyses show that BTX affects mainly those salivary gland acinar cells located around the site of injection [7]. BTX mediated salivary gland protection from radioligand toxicity is intended to cover the gland tissue as completely as possible. Therefore, we deliberately chose six injection points for each parotid and three injection points for each submandibular gland and advocate this injection technique for salivary gland treatment, especially with the use of higher BTX-dosages. The long-term safety of local BTX injections has been proven for many years with full recovery of the target glands within around 16 weeks following BTX injection. The prevalence of neutralizing antibodies was not tested in this study but there is clear evidence for the particularly low antigenicity of IncoA even with higher dosages used in patients with spasticity and dystonia as well as in non-neurological indications [14,15,16,17].

Patients included in the present study were heavily pretreated and showed rapid PCa progression after exhausting all established cancer therapies and thus safety as well as tolerability of the gland protective BTX therapy was of primary importance. A recent series of 26 patients treated with Ac-PSMA after failure of Lu-PSMA reported xerostomia in 100% of patients and 23% stopped Ac-PSMA-RT because of intolerable xerostomia following two RT cycles [18]. These results are in line with a previous report of around 66% loss of parotid and submandibular gland function following two unprotected Ac-PSMA RT cycles [3]. By contrast, our preliminary data suggest that high-dose IncoA in combination with transdermal scopolamine provides a clinically relevant SG protection accompanied by good tolerability.

However, the modalities of SG-protective BTX therapies may vary depending on cancer type and prognosis. In differentiated thyroid cancer for example, more than 70% of patients require only up to two ^131^I radioligand treatment sessions to achieve long-term remission and survival [5]. Whether such a patient group with excellent oncological long-term prognosis or PCa patients in earlier lines of PSMA-therapy might benefit from modified BTX treatment regimen in combination with systemic anticholinergics with the objective to protect all four major SG from radioligand toxicity remains to be examined in future studies.

Interestingly, the strong PSMA radioligand accumulation in salivary glands does not correspond to high PSMA expression levels in the glands, indicating unspecific PSMA radioligand uptake into SG [19]. BTX-A induces reversible cellular changes in salivary glands with decreased mucosal and serous acini diameter and temporary glandular atrophy [8,20]. These effects are probably due to fully reversible glandular denervation resulting in reduced radiation sensitivity [20]. In addition, combined blockage of parasympathetic gland innervation by BTX-A and anticholinergics reduces SG blood flow, resulting in lower accumulation of toxic PSMA and iodine radioligands inside the glands.

In conclusion, salivary gland injections with IncobotulinumtoxinA up to a total dose of 250 units are safe when injected under ultrasound control by an experienced physician, even in seriously ill patients in a fragile medical condition. These novel findings of our preliminary study lay the basis for future trials including botulinum toxin as major component for salivary gland protection in established as well as newly emerging radioligand cancer therapies.

## Figures and Tables

**Table 1:** Botulinum Toxin (IncoA) treatment details and adverse events.

Patient	PG Right IncoA (Units)	SMG Left IncoA (Units)	Total Dose IncoA (Units)	Adverse Event	AE Severity
1	100	50	150	Painful swallowing	Mild
2	100	50	150	-	-
3	120	70	190	Dry mouth	Mild
4	120	70	190	-	-
5	130	70	200	Dry mouth	Mild
6	170	80	250	Dry mouth *	Moderate *
7	170	80	250	Dry mouth	Mild
8	170	80	250	Dry mouth *	Moderate *
9	170	80	250	Dry mouth	Mild
10	170	80	250	Dry mouth	Mild

PG = Parotid gland, SMG = Submandibular gland, * moderate pre-existing xerostomia.

**Table 2:** Concomitant permanent medication.

Any Drug Treatment	*N* = 10
Antiandrogenes	*N* = 5
GnRH-analogues	*N* = 5
Steroids	*N* = 4
RANK-ligand-Inhibitors	*N* = 5
Bisphosphonates	*N* = 1
Opioids	*N* = 6
NSAIDs/COX-2 inhibitors	*N* = 9
Gabapentinoids	*N* = 3
Antihypertensives	*N* = 5
Statins	*N* = 1
Erythropoietin	*N* = 1
Antacids	*N* = 1

## Data Availability

Data available on request due to restrictions e.g. privacy or ethical. The data presented in this study are available on request from the corresponding author.
